# Nasopharyngeal carriage of *Streptococcus pneumoniae* in children under 5 years of age before introduction of pneumococcal vaccine (PCV10) in urban and rural districts in Pakistan

**DOI:** 10.1186/s12879-018-3608-5

**Published:** 2018-12-18

**Authors:** Muhammad Imran Nisar, Kanwal Nayani, Tauseef Akhund, Atif Riaz, Omer Irfan, Sadia Shakoor, Sehrish Muneer, Sana Muslim, Aneeta Hotwani, Furqan Kabir, Cynthia Whitney, Lindsay Kim, Velusamy Srinivasan, Asad Ali, Anita K. M. Zaidi, Fyezah Jehan

**Affiliations:** 1 Department of Pediatrics and Child Health, Aga Khan University, Stadium Road, Karachi, 74800 Pakistan; 2 Centre for Disease Control and Prevention, Atlanta, USA; 3 Bill & Melinda Gates Foundation, Seattle, USA

**Keywords:** PCV10, Pakistan, Introduction, Pneumococcal carriage

## Abstract

**Background:**

Benefits of pneumococcal conjugate vaccine programs have been linked to the vaccine’s ability to disrupt nasopharyngeal carriage and transmission. The 10-valent pneumococcal vaccine (PCV10) was included in the Expanded Program on Immunization (EPI) in Sindh, Pakistan in February 2013. This study was carried out immediately before PCV10 introduction to establish baseline pneumococcal carriage and prevalent serotypes in young children and to determine if carriage differed in urban and rural communities.

**Methods:**

Nasopharyngeal specimens were collected from a random sample of children 3-11 and 12-59 months of age in an urban community (Karachi) and children 3-11 months of age in a rural community (Matiari). Samples were processed in a research laboratory in Karachi. Samples were transported in STGG media, enriched in Todd Hewitt broth, rabbit serum and yeast extract, cultured on 5% sheep blood agar, and serotyped using the CDC standardized sequential multiplex PCR assay. Serotypes were categorized into PCV10-type and non-vaccine types.

**Results:**

We enrolled 670 children. Pneumococci were detected in 73.6% and 79.5 % of children in the infant group in Karachi and Matiari, respectively, and 78.2% of children 12 to 59 months of age in Karachi. In infants, 38.9% and 33.5% of those carrying pneumococci in Karachi and Matiari, respectively, had PCV10 types. In the older age group in Karachi, the proportion was 30.7%, not significantly different from infants. The most common serotypes were 6A, 23F, 19A, 6B and 19F.

**Conclusion:**

We found that about 3 of 4 children carried pneumococci, and this figure did not vary with age group or urban or rural residence. Planned annual surveys in the same communities will inform change in carriage of PCV10 serotype pneumococci after the introduction and uptake of PCV10 in these communities

**Electronic supplementary material:**

The online version of this article (10.1186/s12879-018-3608-5) contains supplementary material, which is available to authorized users.

## Background

The World Health Organization estimates that pneumococcal infections kill around 541,000 children under the age of 5 years globally every year, with pneumococcal pneumonia responsible for 485,000 deaths [1].

The upper respiratory tract is the ecological niche for the pneumococcus and colonization is established during the first months of life [2]. Colonization is generally asymptomatic, but in some it may progress to invasive pneumococcal disease (IPD) or non-invasive disease [3]. Fortunately, both colonization and disease due to *Streptococcus pneumoniae* can be prevented by administration of pneumococcal conjugate vaccine (PCV). Studies done in the US, Africa and Europe have shown that PCV introduction caused a decrease in invasive pneumococcal disease and other pneumococcal syndromes [2, 4–8]. Reports also illustrated that PCV use decreased nasopharyngeal carriage of vaccine serotypes of *Streptococcus pneumoniae* [9–12].The initial vaccine introduced in the immunization programs of most countries in the mid-2000’s was the 7-valent vaccine, which was replaced by the 10- (PCV10) or 13-valent vaccine (PCV13) at the end of the decade [2, 5].

Introduction of PCV10 into routine immunization began in Sindh, Pakistan in February 2013 with financing assistance from the GAVI Alliance. The vaccine was introduced with a 3+0 schedule, with doses given at 6, 10 and 14 weeks of life. No catch-up vaccination was offered.

The prevalence of pneumococcal carriage can be used to estimate the potential for PCVs to reduce transmission of vaccine-type strains [13] and indirectly translates into impact on disease, as shown by previous carriage studies [11, 14, 15]. Such knowledge of baseline serotype prevalence among carriage strains in young children is important for countries like Pakistan, where the vaccine coverage is low and incidence of invasive pneumococcal disease (IPD) is quite high [16, 17]. There could be possible differences between carriage rates in the urban and rural populations due to difference in accessibility to care, socio-economic conditions and public health awareness. It would be beneficial to study both populations separately, in order to come up with more effective health policies for each sector.

We undertook this study to determine baseline carriage rates in children 3 to 11 months of age in urban and rural communities and 1-5 years old in the urban community of Sindh, Pakistan, before introduction of PCV10.

## Methods

### Study design and setting

This was a cross-sectional survey done in two union councils of rural Matiari and one union council of urban Karachi, both in the southern province of Sindh, Pakistan. These sites were selected because of pre-existing infrastructure and ongoing research in the community. A union council is the smallest administrative unit in the government structure. Matiari is around 180 km away from Aga Khan University (AKU) in Karachi, which houses the research unit and the Infectious Disease Research Laboratory (IDRL). The field site in urban Karachi was located around 20 km from AKU. This survey was done during the months of January and February 2013, immediately before the introduction of PCV10 vaccine in Sindh.

All children aged 3 to 11 months resident in the study area in the Matiari site and all children under the age of 5 years in the Karachi site were eligible to be enrolled in the study. A random sample of children 3 to 11 months of age was selected from a household listing of two union councils, Khyber and Seekhat, in Matiari. In Karachi an additional random sample of children 12 to 59 months was selected from a line listing of all children living in the area. Children with a history of severe acute respiratory illness in the last 2 weeks, presence of chest wall indrawing, blue skin discoloration (cyanosis) and fast breathing were excluded from the study. Children with moderate to severe cerebral palsy, neurological disorders affecting swallowing and nose and throat disorders were also excluded. If a child was not found, or found to be ineligible in the random list, the next eligible child in the list was approached.

Baseline demographic data and clinical characteristics of children were collected through a questionnaire (available as a supplementary file). The questionnaire was developed, translated and back-translated between English, Urdu and Sindhi languages and pretested in the field before implementation. A two-day training was held for staff involved in the study to standardize data and specimen collection methods. Crowding index was developed by dividing number of people by number of rooms in the household, axillary temperature was measured using a digital thermometer and vaccination history was collected either verbally or verified through card.

### Laboratory methods

Nasopharyngeal swabs were collected by trained staff as per World Health Organization methods [18]. Swabs were immediately placed into skim milk tryptone glucose glycerol (STGG) media in cryovials and transported to the Infectious Disease Research Laboratory (IDRL) in Karachi at 2-8°C within 6-8 hours of collection. Upon reaching the central laboratory, inoculated STGG was vortexed for 10-20 seconds to disperse organisms from the swab and frozen in an upright position at -70°C until further processing. Samples were then cultured in batches of 20-40 samples and sub-cultured as described in the CDC protocol (https://www.cdc.gov/streplab/downloads/pcr-pneumo-carriage-march2010.pdf). Briefly, an aliquot of the thawed STGG mixture was enriched in Todd Hewitt broth supplemented with rabbit serum (20%) and yeast extract (0.5%) and subcultured onto TSA medium with 5% sheep blood. After 18-24 hours of incubation, plates were examined for the presence of alpha-haemolytic colonies with morphological characteristic features of pneumococci. There was only one case where two colonies were identified and the dominant one was elected. Identification was confirmed by susceptibility to Optochin and bile solubility testing.

Serotypes for the pneumococcal isolates were deduced by sequential conventional multiplex PCR method as described in CDC Streptococcus laboratory protocols [19]. Briefly, DNA was extracted by boiling a loop full of bacterial culture in 1 ml of TE Buffer at 100 °C for 10 minutes followed by centrifugation at 10,000 rpm for 10 minutes; the resulting supernatant was collected in a new sterile 1.5 ml microfuge tube and 2 μl of the extracted DNA was directly used in the PCR reaction. The *cpsA* gene was included in each multiplex reaction to serve as an internal positive control. PCR master mix (of 23 μl) was prepared using 25 μM working stock of primers, 2X Qiagen multiplex PCR buffer, Qiagen Q solution and nuclease free water, with 2 μl of DNA template added to the reaction mixture. Amplification was carried out in an Eppendorf Master Cycler Gradient with the following temperature profile: initial denaturation at 95 °C for 15 minutes, then 35 cycles consisting of 94 °C for 30 seconds, 54 °C for 90 seconds, and 72 °C for 60 seconds, followed by a final extension step at 72 °C for 10 minutes. The amplified PCR products were separated on 2% (w/v) agarose gel electrophoresis, stained with 1X SYBR-green staining solution, and documented under BioRad Gel Doc imager. Serotypes detected through sequential multiplex conventional PCR were further confirmed by monoplex PCR, using the same PCR conditions, to avoid misidentification due to non-specific bands [20].

Serogroup 6 was further discriminated into individual serotypes, 6A, 6B, 6C, and 6D by conventional PCR as described by Jin et al. [19, 20] with slight modifications, using 25 μM working stocks of primers, 2X Qiagen multiplex PCR buffer, Qiagen Q solution and nuclease free water, with 2 μl of DNA template added to the reaction mixture. Amplification reactions were conducted using a Eppendorf Master Cycler Gradient with the following temperature profile: initial denaturation at 95°C for 15 minutes, then 35 cycles consisting of 94°C for 30 seconds, 66.2°C for 60 seconds, and 72°C for 60 seconds, followed by a final extension step at 72°C for 10 minutes. Appropriate pneumococcal serotype controls obtained from the CDC streptococcal lab were included in every reaction. The amplified PCR products were separated on 2.5% (w/v) agarose gel electrophoresis, stained with 1X SYBR -green staining solution, and documented under a Biorad Gel Doc imager.

Identification of non-typeable pneumococcal isolates (those with no positive results for any tested serogroups/types) was confirmed with a *lytA* real time PCR as described by Carvalho et al. [21].

For Optochin susceptibility and bile solubility reactions, the following reference American Type Culture Collection (ATCC) strains were used for quality control: *Streptococcus pneumoniae* ATCC 49619 and *Enterococcus faecalis* ATCC 29212. In addition, a total of 176 randomly selected pneumococcal isolates were sent to CDC to cross check PCR serotying results with the Quellung reaction; 95% concordance was found between PCR and CDC Quellung reaction results.

### Sample size calculations

Sample size was calculated based on a predictive decline in NP carriage in subsequent years after the introduction of vaccine. There are no recent data regarding prevalence of pneumococcal carriage in Pakistan. One study from 1993 reported prevalence of PCV7 serotypes in children 2-24 months as 50% and 65 % [22]. To obtain maximum sample size, a baseline carriage rate of 50% was assumed. This gave a sample size of 220 in order to show a decrease in prevalence from 0.5 to 0.3 after introduction of PCV10 with 80% power and 5 % level of significance.

### Data management and quality assurance

Data consisting of demographic and socio-economic indicators as well as general health status and common symptoms were collected on paper forms by trained personnel at the designated fieldwork sites. All participants were assigned a unique identifier and a linking identification number from the demographic surveillance system was also noted. All completed forms were reviewed by the study supervisor at the end of the day for completeness and consistency. Data were dual entered in an MS Access database and validation and consistency checks were run. Analysis was done using STATA version 12 and MS EXCEL. Confidence bound for predictors such as age, gender, educational status etc. were created using a logistic regression model. All variables that were found to be significant at a *p*-value of ≤ 0.10 at univariate level were added in a multivariate model. A stepwise backward elimination approach was used to derive a parsimonious model retaining only variables with a *P*-value of <0.05. Odds ratios with 95% confidence intervals were calculated. Analysis was done looking at overall nasopharyngeal carriage as well as carriage of PCV10 specific serotypes as the outcome and 95% confidence intervals were presented.

#### Ethical approval

Ethical approval was obtained from Aga Khan University’s Ethical Review Committee. The study was performed according to approved protocol.

## Results

A total of 220 and 225 children aged 3 to 11 months old were enrolled in Karachi and Matiari, respectively. An additional 225 children aged 12 to 59 months were enrolled in Karachi. Table 1 gives the demographic and clinical characteristics of all enrolled children.

**Table 1:** Socio demographic and clinical characteristics of enrolled children

Variable	Karachi (3 -11 months) *N* = 220	Karachi (12-59 months) *N* = 225	Matiari (3 -11 months) *N* = 225
	N (%)	N (%)	N (%)
Age, mean (SD)	9.8 (6.1)	39.6 (12.7)	6.5 (2.6)
Male gender	102 (46.4)	107 (47.6)	125 (55.6)
Primary caretaker education			
Illiterate	92 (41.9)	103 (45.8)	144 (64.0)
1-5 years	25 (11.4)	19 (8.4)	31 (13.8)
6-10 years	47 (21.4)	43 (19.1)	16 (7.1)
>10 years	56 (25.5)	60 (26.7)	34 (15.1)
Crowding index (persons/room)			
Mean (SD)	4.4 (2.0)	4.4 (2.1)	5.8 (2.5)
Hospitalization in last year	15 (6.8)	0 (0.0)	8 (3.6)
No. of outpatient visits in last month			
Mean (SD)	1.8 (1.0)	1.7 (0.9)	2.0 (2.5)
Fever (current or in last 3 days)	30 (13.6)	20 (8.9)	4 (1.8)
Cough	64 (29.1)	51 (22.7)	41 (18.2)
Type of Fuel used in cooking			
Natural gas	216 (98.2)	220 (97.8)	28 (12.4)
Other	4 (1.8)	5 (2.2)	197 (87.6)
Child exposed to smoke during cooking	0 (0.0)	1 (0.4)	150 (66.7)
Smoker in the Household	48 (21.8)	45 (20.0)	110 (48.9)
Ever vaccinated^a^	170 (77.3)	170 (75.6)	157 (69.8)

*SD* Standard Deviation ^a^having received any vaccine

It was noted that the literacy rate among the primary caretakers in the Matiari site was lower than that in Karachi, and the household crowding index was higher. A huge majority of children living in Matiari were exposed to smoke, through cooking fuel as well as passive tobacco smoking. Immunization rates were low in both sites.

Overall carriage did not differ significantly by age or between the urban and rural sites (Table 2).

**Table 2:** Number and proportion of children carrying pneumococcus and vaccine serotypes by age and study site

Study Site	Number of participants (n)	Number culture-positive for pneumococcus (%)	Number with PCV10 serotypes (% of isolates)	Number with PCV13 serotypes (% of isolates)
Matiari (3-11 months)	225	179 (79.5)	60 (33.5)	95 (53.1)
Karachi (3-11 months)	220	162 (73.6)	63 (38.9)	88 (54.3)
Karachi (12-59 months)	225	176 (78.2)	54 (30.7)	78 (44.3)

In the infant group, 73.6% (95% CI, 67.3-79.3) and 79.5% (95% CI, 73.7-84.6) of the nasopharyngeal specimens were positive for pneumococci in Karachi and Matiari, respectively. In the older age group in Karachi, 78.2% (95% CI, 72.2-83.4) of specimens were positive for pneumococci. Among the isolates, the proportion that was PCV10 serotypes was 38.9% (95% CI, 31.3-46.8) and 33.5% (95% CI, 26.6-41.0) in 3 to 11 months-old children in Karachi and Matiari, respectively, and 30.7% (95% CI, 24.0-38.0) in children 12-59 months of age in Karachi (Table 2).

There was no statistically significant difference in carriage between urban and rural populations in the infant group or in the two age groups in the urban population, for both vaccine and non-vaccine types. Taking all sampled children as the denominator, the carriage rate for PCV10 serotypes was 26.7% and 28.6% for children 3-11 months in Matiari and Karachi, respectively, and 24% in children 12-59 months in Karachi. Preliminary results have been previously presented as an abstract [23].

**Fig. 1:**
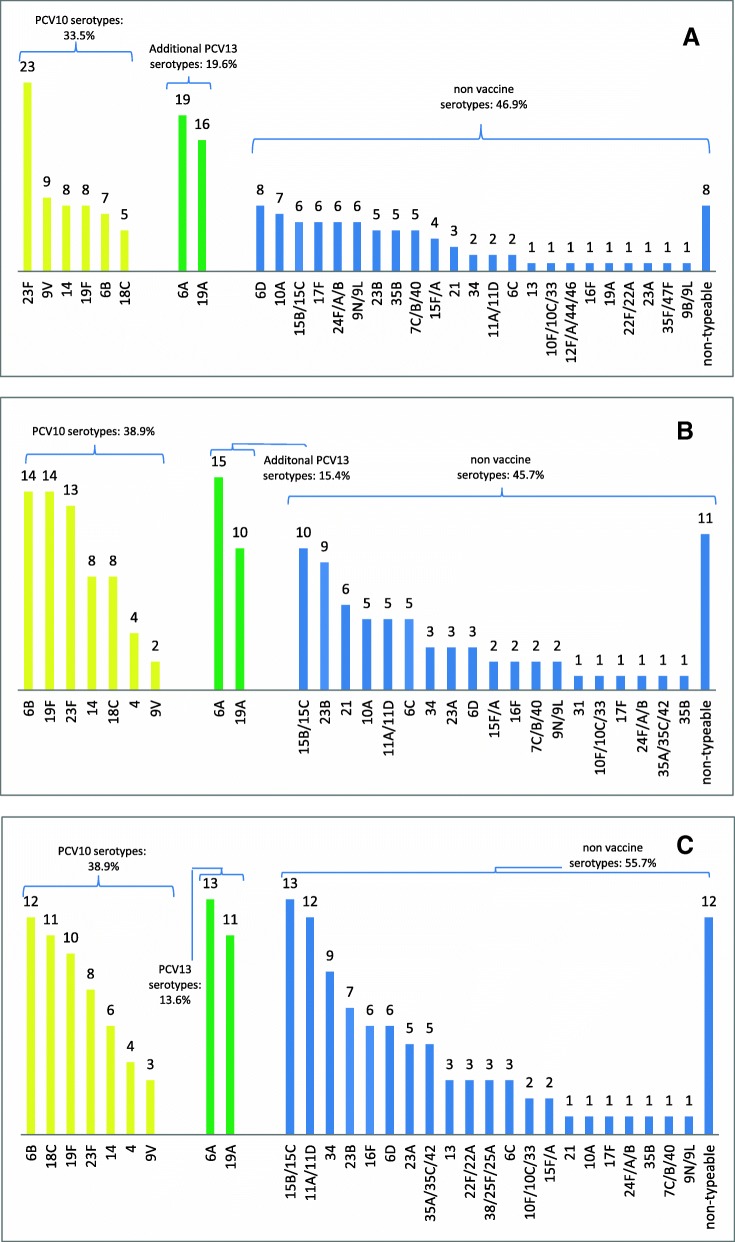
Number of serotypes by study site and age group; **a**) Matiari (rural site) children 3-11 months of age; **b**) Karachi (urban site) children 3-11 months of age; and **c**) Karachi (urban site) children 12-59 months of age

Figure 1 gives the serotype distribution of the specimens that were culture positive in Karachi and Matiari. The most frequent PCV10 serotypes in Karachi 3-11 month-olds were 6B, 19F and 23F, while in Matiari 23F, 9V and 14 were most common. In the older age group in Karachi, the most common serotypes were 6B, 18C and 19F. Carriage rates for PCV13-specific serotypes were significantly higher than PCV10 serotype carriage in Matiari (53.1% vs. 33.5%, *p*-value<0.001), Karachi 3-11 month-olds (54.3% vs. 38.9%, *p*-value=0.005) and Karachi children 12-59 months (44.3% vs. 30.7%, *p*-value=0.008; Table 2). The carriage rate for PCV10 as well as 6A was 44.1% in Matiari 3-11 months, 48.1% in Karachi 3-11 months and 38.1% in Karachi 12-59 months. None of the socio-demographic or clinical features we measured were associated with pneumococcal carriage overall or with carriage of PCV10 serotypes (Additional file 1: Table S1)

## Discussion

This study has revealed high point prevalence of pneumococcal carriage in the nasopharynx among children in lower Sindh. These rates are higher than those observed in other countries in the region [21]. Carriage rates were closer to those reported in many African countries and rural areas in other parts of the world [24–26]. Pneumococcal carriage in the urban area was as high as in the rural setting. This is in contrast to other studies which have shown the carriage in urban areas to be lower [27, 28]. Our results further reveal that there is no variation in the carriage rates or carriage of vaccine serotypes across the two age groups we examined, in spite of notable differences in smoke exposure and markers of economic status such as maternal education level between urban and rural areas.

Our results have shown a significant burden of PCV13-specific serotypes among children in Pakistan. Although it has been proposed that PCV10 confers cross-protection against some PCV13 specific serotypes such as 6A and 19A, the evidence regarding this is inconclusive [29, 30]. Disease surveillance data will be needed to understand if the additional serotypes covered by PCV13 cause enough burden to warrant a change in the pneumococcal vaccine used in Pakistan’s immunization program. This study indicates that there is a large reservoir of pneumococci in the nasopharynx in infants, implying an effective role for this vaccine in this age group in both urban and rural areas. Thus it will be important to see the impact of the vaccine on carriage rates with the current EPI program, especially because coverage rates for routine infant immunizations are low in some areas of Pakistan [17]. While older children may be less susceptible to disease than infants, our data showing high carriage rates suggests these older children may be functioning as direct reservoirs for transmitting pneumococci to each other, to adults, and to the more susceptible infants.

An important strength of the study is that it was done immediately before the introduction of PCV10 in Pakistan. None of the children in the study area had received a pneumococcal vaccine. We intend to repeat similar cross sectional surveys in subsequent years after the introduction of vaccine to gauge the effect of the PCV10 vaccine in decreasing carriage of vaccine type strains and whether particular non-vaccine serotype pneumococci become more prevalent. We also wish to study whether the change in carriage led to a change in the prevalence of IPD and the burden of pneumonia.

There are some limitations to the study. The study areas were limited to an urban and rural population in the southern province of Pakistan, and whether carriage or serotype distribution is different in other parts of Pakistan is unknown. Although we tried to ensure quality control by sharing a subset of isolates with CDC and comparing with Quellung method, PCR is still not as sensitive and we may have underestimated carriage [31]. Another limitation might be that we did not evaluate carriage density among carriers of pneumococci, because we opted to use culture methods that included an enrichment step designed to optimize recovery of pneumococci; therefore, we were not able to examine the association between carriage density and variables such as fever, hospitalization, and exposure to smoke. We were also unable to identify known risk factors associated with carriage such as breastfeeding and prior antibiotic use because this information was not collected. This may explain why we found no risk factors.

Since carriage density varies with age, the results might have been significantly different across age groups [32]. Since we studied the prevalence of vaccine and non-vaccine serotypes, the main crux of this study is therefore to inform serotype distribution among carriage pneumococci in a vaccine-naïve pediatric population, and serve as a baseline for assessing impact of PCV10 introduction on carriage rates among vaccinees as well as non-vaccinated older children (indirect effect).

## Conclusion

This survey establishes the pre PCV10 introduction vaccine and non-vaccine serotype carriage rate in children in a rural and urban community in Pakistan. In spite of differences in the social situation of children enrolled from these two communities, carriage overall and of vaccine serotypes was equally high in both settings, suggesting potential benefits from PCV introduction regardless of setting. Annually planned surveys in the same communities will inform change in carriage rate after the introduction and uptake of PCV10 in these communities. Linkage with data showing impact on disease endpoints will allow for a full picture of the impact of PCV10 in Pakistan.

## Additional file

Additional file 1: **Table S1.** Association of different variables with pneumococcal carriage. (DOC 47 kb)

## Abbreviations

AKU: Aga Khan University
CDC: Centre for Disease Control
GAVI: Global Alliance for Vaccine Initiatives
IDRL: Infectious Disease Research Laboratory
IPD: Invasive Pneumococcal Disease
PCR: Polymerase Chain Reaction
PCV: Pneumococcal Conjugate Vaccine
PCV10: 10Valent Pneumococcal Conjugate Vaccine
PCV13: 13Valent Pneumococcal Conjugate Vaccine
PCV7: 7Valent Pneumococcal Conjugate Vaccine
STGG: Skim Milk Tryptone Glucose Glycerol
TSA: Tryptic Soy Agar
