# Correction to: Nasopharyngeal carriage of *Streptococcus pneumoniae* in children under 5 years of age before introduction of pneumococcal vaccine (PCV10) in urban and rural districts in Pakistan

**DOI:** 10.1186/s12879-019-3733-9

**Published:** 2019-02-05

**Authors:** Muhammad Imran Nisar, Kanwal Nayani, Tauseef Akhund, Atif Riaz, Omar Irfan, Sadia Shakoor, Sehrish Muneer, Sana Muslim, Aneeta Hotwani, Furqan Kabir, Cynthia Whitney, Lindsay Kim, Velusamy Srinivasan, Asad Ali, Anita K. M. Zaidi, Fyezah Jehan

**Affiliations:** 1 Department of Pediatrics and Child Health, Aga Khan University, Stadium Road, Karachi, 74800 Pakistan; 2 Centre for Disease Control and Prevention, Atlanta, USA; 3 Bill & Melinda Gates Foundation, Seattle, USA

**Correction to:** **BMC Infectious Diseases (2018) 18:672**

**https://doi.org/10.1186/s12879-018-3608-5**

Following publication of the original article [1], the author reported his name has erroneously spelled as Omer Irfan. The correct name is Omar Irfan.

It has been corrected in the original article as well.

The publisher apologizes for any inconvenience caused by this error.

## References

[1] Nisar (2018). BMC Infect Dis.

