# Supplementary material for: Nasopharyngeal carriage of Streptococcus pneumoniae in children under 5 years of age before introduction of pneumococcal vaccine (PCV10) in urban and rural districts in Pakistan
Source: BMC Infect Dis. 2018 Dec 18;18:672. doi: 10.1186/s12879-018-3608-5 (PMC6299586; doi:10.1186/s12879-018-3608-5)
Supplement: Supplementary file 1 — Table S1. Association of different variables with pneumococcal carriage. (DOC 47 kb) [file 12879_2018_3608_MOESM1_ESM.doc]

**Appendix**

**Table 1- Association of different variables with pneumococcal carriage**

|  | Culture positive as the outcome, (n=670) | PCV 10 serotypes as the outcome, (n=531) |
| --- | --- | --- |
| Age | OR (95%CI) | OR (95%CI) |
| ≤ 12 months | Ref | Ref |
| > 12 months | 1.2(0.8-1.7) | 0.8(0.5-1.1) |
| Sex |  |  |
| Male | Ref | Ref |
| Female | 1.1(0.7-1.6) | 0.9(0.6-1.3) |
| Education of primary wage earner |  |  |
| No education | Ref | Ref |
| 1-5 years | 0.7(0.4-1.2) | 0.51(0.3-0.99) |
| 6-16 years | 0.8(0.5-1.2) | 0.76(0.5-1.1) |
| Crowding index | 1.0(0.9-1.1) | 1.0(0.9-1.1) |
| Hospitalization in previous year |  |  |
| No | Ref |  |
| Yes | 0.7(0.3-1.8) | 1.1(0.4-3.0) |
| No. of outpatient visits in last month | 0.9(0.8-1.0) | 1.1(0.9-1.2) |
| Fever |  |  |
| No | Ref | Ref |
| Yes | 0.8(0.4-1.5) | 1.2(0.6-2.2) |
| Cough |  |  |
| No | Ref | Ref |
| Yes | 0.9(0.6-1.4) | 0.8(0.5-1.2) |
| Smoker in Household |  |  |
| No | Ref | Ref |
| Yes | 1.0(0.7-1.5) | 1.0(0.7-1.5) |
| Fuel used for cooking |  |  |
| Natural Gas | Ref | Ref |
| Other (wood , crops, dung) | 1.2(0.8-1.7) | 1.0(0.7-1.4) |
| Child exposed to smoke during cooking |  |  |
| No | Ref | Ref |
| Yes | 1.0(0.6-1.6) | 1.0(0.7-1.6) |
| Ever Vaccinated |  |  |
| Yes | Ref | Ref |
| No | 1.2(0.7-1.8) | 1.2(0.8-1.8) |
